# SIRT1 inhibits monocyte adhesion to the vascular endothelium by suppressing Mac-1 expression on monocytes

**DOI:** 10.1038/s12276-019-0239-x

**Published:** 2019-04-25

**Authors:** Seung Jin Lee, Seung Eun Baek, Min A. Jang, Chi Dae Kim

**Affiliations:** 1 College of Pharmacy, Pusan National University, Busan, 46241 Republic of Korea; 2 Department of Pharmacology, School of Medicine and Gene & Cell Therapy Research Center for Vessel-associated Diseases, Pusan National University, Gyeongnam, 50612 Republic of Korea

**Keywords:** Mechanisms of disease, Lipid signalling, Transcriptional regulatory elements, Chronic inflammation, Atherosclerosis

## Abstract

SIRT1 signaling pathways modulate vascular inflammation; however, the precise role of SIRT1 in monocyte adhesion to the vascular endothelium, a key event initiating vascular inflammation, is unclear. Thus, this study investigated the roles and molecular interaction of SIRT1 and TLR2 in regulating monocyte adhesion to the vascular endothelium. In vitro, both Mac-1 expression and the endothelial adhesion of THP-1 cells stimulated with Pam3CSK4, a TLR2 ligand, were markedly increased in association with a decreased expression of SIRT1. In THP-1 cells stimulated with Pam3CSK4, the promoter activity and expression of SIRT1 were decreased. The TLR2-dependent suppression of SIRT1 expression in THP-1 cells was mediated by the transcription factors NF-κB and CREB, suggesting that the TLR2-mediated NF-κB and CREB signaling downregulated SIRT1 expression in monocytes. In peripheral blood monocytes (PBMCs) isolated from SIRT1 transgenic (TG) mice and THP-1 cells treated with recombinant SIRT1, both the increased Mac-1 expression and endothelial adhesion induced by Pam3CSK4 were significantly attenuated. In addition, the *en face* immunohistochemical study showed a marked increase in monocyte adhesion to the aortic endothelium of WT mice treated with Pam3CSK4, which was significantly attenuated in Pam3CSK4-treated SIRT1 TG mice. Moreover, a greater number of atherosclerotic plaques formed in WT mice fed a high-fat diet than in SIRT1 TG mice, indicating a pivotal role for SIRT1 in preventing vascular inflammation. Based on these results, SIRT1 might be a potential target for researchers aiming to develop therapeutic interventions for vascular inflammation, including atherosclerosis.

## Atherosclerosis: Intervening in the early stages of plaque formation

Researchers have identified a possible new tool, the signaling molecule SIRT1, to fight vascular diseases such as atherosclerosis, hardening of the arteries. In atherosclerosis, plaque builds up in arteries, restricting blood flow and potentially causing heart attack or stroke. Early in the disease, white blood cells called monocytes stick to artery walls, triggering inflammation. Administering SIRT1 was known to decrease inflammation, but how it did so was unclear. Chi Dae Kim at Pusan National University in South Korea and co-workers investigated the signals that trigger monocytes to attach to artery walls, and how SIRT1 might prevent attachment. After identifying the signals, further testing in mice fed a high-fat diet showed that SIRT1 strongly decreased plaque formation. These findings illuminate the causes and progression of atherosclerosis and may help identify better treatments.

## Introduction

Monocytes play an essential role in the progression of vascular inflammation, including atherosclerosis^1,2^. Because monocyte adhesion to the endothelium in blood vessels is an essential event in vascular inflammation^3^, circulating monocytes are critically involved in the progression of atherosclerosis^4,5^. The adhesion of monocytes to the arterial endothelium and their subsequent migration into the subendothelial region is a hallmark of vascular inflammation^6,7^. Although monocyte adhesion to endothelial cells, which depends on the interaction between adhesion molecules on monocytes and endothelial cells, represents a key stage in modulating vascular inflammation^8,9^, the effective regulators of monocyte adhesion to the vascular endothelium have not been identified.

Toll-like receptors (TLRs) expressed at high levels in most inflammatory cells are activated by pathogens and endogenous ligands, and are important for the induction of innate immune responses^10,11^. Among the various TLRs, TLR2 and TLR4 are involved in the initiation and progression of vascular inflammation, including atherosclerotic diseases^12^. In particular, TLR2 levels are markedly increased in human atherosclerotic plaques^13,14^ and murine models of atherosclerosis^15,16^, and stimulation of TLR2 with Pam3CSK4 induces the production of pro-inflammatory cytokines^17,18^. In addition, atherosclerosis was ameliorated in TLR2 knockout mice, suggesting that TLR2 plays a critical role in the progression of vascular inflammation. Although monocyte SIRT1 overexpression has been shown to prevent vascular inflammation by improving vascular functions^19,20^, the precise role of SIRT1 in TLR2-induced vascular inflammation has not been determined.

Recent studies examining the vascular biology and inflammation have reported the protective effects of SIRT1 on vascular inflammation^21,22^. SIRT1 exerts anti-inflammatory effects on monocytes, which are attributed to the downregulated expression of various pro-inflammatory cytokines^23^. Thus, SIRT1 represents a promising target for the development of therapeutic interventions for vascular inflammation. Reportedly, various transcription factors responsible for inducing SIRT1 expression, including nuclear factor κB (NF-κB) and cAMP response element-binding protein (CREB), have been characterized^24^. Many of these factors are known to be critically involved in the mechanism regulating metabolism and have been shown to participate in vascular inflammation. Because multiple factors regulating SIRT1 probably play important roles in vascular inflammation, NF-κB and CREB are not likely to be the exclusive factors responsible for the preventing vascular inflammation.

Although several studies have reported that SIRT1 signaling pathways modulate vascular inflammation, the molecular interaction between SIRT1 and TLR2 in the progression of vascular inflammation remains unclear. In this study, we investigated the role of SIRT1 in TLR2-mediated monocyte adhesion to the vascular endothelium, a key early event in the progression of vascular inflammation. Moreover, the molecular mechanisms connecting TLR2 and SIRT1 expression in monocytes were also investigated.

## Materials and methods

### Chemicals and antibodies

Pam3CSK4 was purchased from InvivoGen Co. (San Diego, CA). The anti-human Mac-1 antibody (cat no. 16-0113) and anti-mouse IgG isotype control antibody (cat no. 16-4714) were purchased from eBioscience (San Diego, CA). The R-phycoerythrin (PE)-conjugated mouse anti-human Mac-1 (clone ICRF44; BD) antibody (cat no. 555388) and PE-conjugated mouse IgG isotype control (clone MOPC-21) antibody (cat no. 555749) were obtained from BD (San Diego, CA). Calcein AM (an acetomethoxy (AM) derivative of calcein) and luciferase reporter constructs containing NF-κB (cat no. 219078) and CREB (cat no. 219076) consensus-binding sites were purchased from Agilent Technologies (Santa Clara, CA, USA). The anti-SIRT1 antibody and horseradish peroxidase (HRP)-conjugated IgG (secondary antibody) were obtained from Santa Cruz Biotechnology Inc. (Beverly, MA). ImProm-II Reverse Transcription Kits were supplied by the Promega Corporation (Madison, WI). DNeasy Tissue Kits and QIAprep Spin Kits were purchased from Qiagen (GmbH, Germany).

### Animals

SIRT1-transgenic (TG) mice were kindly provided by Dr. J.W. Park (Seoul National University, Korea) and were established with a Myc-His-tagged SIRT1 plasmid containing a CMV promoter. Detailed information has been provided in a previous study^25^. Wild-type (WT) C57BL/6N mice were purchased from Jackson Laboratories (Harlan Nossan, Italy). Tails were clipped at 2–3 weeks of age, and genomic DNA was isolated for genotyping. Mice were fed a high-fat diet (HFD; 45% kcal from fat, Harland Tekland, Madison, WI, USA) for 12 weeks to induce atherosclerosis as a model of vascular inflammation. All animal procedures were performed in accordance with the Guide for the Care and Use of Laboratory Animals published by the US National Institutes of Health (NIH Publication No. 85–23, revised 2011), and experimental protocols involving animals were approved by the Institutional Animal Care and Use Committee of Pusan National University (PNU-2016-1310). Animals were housed in an air-conditioned room at 22–25 °C on a 12-h light/dark cycle. Food and water were provided *ad libitum.*

### Cell culture

THP-1 (human monocytic leukemia cell line) cells were purchased from the ATCC (Manassas, VA, USA). Cells were grown in the RPMI 1640 medium (Life Technologies) supplemented with 10% heat-inactivated fetal bovine serum (FBS), an antibiotic–antimycotic solution and L-glutamine (Life Technologies). Human umbilical vein endothelial cells (HUVECs) were obtained from Lonza Walkersville, Inc. (Walkersville, MD) and cultured in the endothelial growth medium-2 (EGM-2 MV, Lonza). Cells were maintained at 37 °C in a humidified 5% CO_2_/95% air atmosphere.

### Isolation of peripheral blood mononuclear cells

Blood was obtained from Pam3CSK4-treated WT and SIRT1 TG mice (8-week-old, 19–22 g) killed by cervical dislocation by puncturing the heart and collecting the blood in 2 mM EDTA vacutainer tubes (Sarstedt, Newton, NC). Age-matched WT and SIRT1 TG mice were used as control groups. Peripheral blood mononuclear cells (PBMCs) were separated by density-gradient centrifugation using a Histopaque®-1077 (Sigma) separating solution (Biochrom, Berlin, Germany)^26^. The cell layer was collected, washed twice with Dulbecco’s PBS, and resuspended in the DMEM (GIBCO 41966) containing 10% heat-inactivated FBS (Biochrom, Berlin, Germany) and 1% penicillin–streptomycin (Sigma-Aldrich Inc., St. Louis, MO).

### Adhesion assay

THP-1 cells and mouse PBMCs were labeled with 0.2 mg/L calcein-AM for 30 min at 37 °C, and seeded onto 90% confluent HUVECs. After 2 h, co-cultured cells were washed with 1× PBS containing 1% bovine serum albumin (BSA), and images were obtained using an inverted optical microscope (Axiovert 25) and Axio Vision Release 4.7 software (Carl Zeiss MicroImaging GmbH, Oberkochen, Germany). Locations were quantified using a Metamorph image analysis system (Molecular Devices, LLC, Downingtown, PA, USA).

### RNA isolation and real-time PCR

Mac-1 mRNA expression was quantified by real-time PCR using GAPDH mRNA as the internal standard. The total RNA was isolated with TRIzol reagent (Life Technologies), and the cDNA templates were synthesized using the Improm-II reverse transcription system, according to the manufacturer’s instructions. The synthesized cDNAs were used as templates for real-time PCR, which was performed using SYBR green real-time master mix (Geneall, Seoul, Korea). The cDNA templates were amplified with real-time PCR using specific primers for Mac-1 (forward, 5′-CAG CCT TTG ACC TTA TGT CAT GG-3′ reverse, 5′-CCT GTG CTG TAG TCG CAC T-3′) and GAPDH (forward, 5′-GAG TCA ACG GAT TTG GTC GT-3′ reverse, 5′-TTG ATT TTG GAG GGA TCT CG-3′).

### Flow-cytometry analysis

Cells were collected from cultures and washed with fluorescence-activated cell sorting (FACS) buffer (PBS containing 1% FCS and 0.05% NaN_3_). Cells were then incubated with an FcR blocker (anti-human IgG; Sigma-Aldrich Co.) to block nonspecific antibody binding, and incubated with a PE-conjugated mouse anti-human Mac-1 antibody (clone ICRF44; BD) with matched pairs of isotype control antibodies. The cells were analyzed using a FACSCalibur flow cytometer and CELLQUESTPRO software (BD).

### Western blot analysis

Cells were washed with ice-cold PBS and lysed in lysis buffer to assess SIRT1 levels. Whole-cell lysates were collected, and protein concentrations were determined with bicinchoninic acid kits (Sigma-Aldrich Co.). Cellular proteins were resolved using 10% sodium dodecyl sulfate-polyacrylamide gel electrophoresis and transferred to polyvinylidene difluoride membranes (GE Healthcare Bio-Sciences Corp., Piscataway, NJ, USA). After blocking the membranes with 1× TBST containing 5% skim milk, membranes were probed with an anti-SIRT1 (1:1000) or anti-alpha actin antibody (1:10,000). After incubation with secondary antibodies, immune complexes were detected using ECL reagents, according to the manufacturer’s instructions.

### Preparation of promoter constructs

The SIRT1 promoter construct containing 2.3 kb of the 5′-flanking promoter region was kindly provided by Dr. Masafumi Ito (Gifu International Institute of Biotechnology, Japan). Plasmids carrying the different fragments of the 5′-flanking promoter regions were created by PCR cloning using specific forward primers (p1425; 5′-AGC TAT TTG TCC TAA TGG TAC CTC-3′, p1030; 5′-AGA AAC GGC TAG GTA CCT CAC GCT AGA-3′, p797; 5′-ACG TCA AAG GTC TTC CCA GGT ACC CAT ATG-3′, p485; 5′-AGC TAA GTC TTA GGT ACC TTC AGC TGT-3′, p278; 5′-AAT TTG GGT ACC CTA CAC GCT CGC-3′), which all contained the *Kpn*I site, and the corresponding downstream primers (5'-GAT ATC CTC GAG CTC GCC TCC TCT GCT-3'). The SIRT1 promoter sequence was analyzed for any transcription factor-binding sites within the 5′-flanking promoter region using the sequence motif search program devised by TFsearch (http://mbs.cbrc.jp/research/db/TFSEARCH.html).

### Transient transfection and the luciferase assay

Cells were transfected with luciferase reporter plasmids using Lipofectamine LTX reagent (Life Technologies), according to the manufacturer’s protocol. Cell lysates were prepared using a passive lysis buffer from a Promega assay system (Promega Corporation) and used to determine luciferase activity with the dual luciferase reporter assay system (Promega Corporation). Transfection efficiencies were compared after normalizing firefly luciferase values to Renilla luciferase values. The results were presented as means ± SEMs of independent experiments performed in triplicate.

### Site-directed mutagenesis

Mutations in the NF-κB and CREB-binding sites in the SIRT1 promoter region of the pGL4-SIRT1 plasmid were generated using a QuikChange site-directed mutagenesis system (Stratagene, CA, USA). The primers used to generate the mutant NF-κB and CREB-binding sites were: NF-κB: wild-type, 5′-GGGAATTCACACA-3′; mutant, 5′-*ATCG*AT*CGAT*ACA-3′. CREB: wild-type, 5′-CACGTCAAAGG-3′; mutant, 5′-C*GTAC*C*T*AAGG-3′. PCR was performed using two antiparallel primers containing the required nucleotide substitutions and the pGL4-SIRT1 plasmid as a template. PCR products were then treated with DpnI endonuclease, and the sequences of mutant constructs were confirmed by performing bi-directional DNA sequencing.

### Chromatin immunoprecipitation assays

Chromatin immunoprecipitation (ChIP) assays were performed using the Millipore ChIP kit (Millipore, Billerica, MA, USA) according to the manufacturer’s instructions, with minor modifications. Briefly, THP-1 cells were inoculated into a 10-cm dish (5 × 10^6^ cells/dish) and fixed with 1% formaldehyde. Cell pellets were resuspended in SDS lysis buffer containing protease inhibitors (1 mM PMSF, 1 mg/mL aprotinin and 1 mg/mL pepstatin A), and the cell suspension was sonicated using a Misonix sonicator 3000 (Misonix, Farmingdale, NY, USA), centrifuged, and diluted 10-fold in ChIP dilution buffer. After removing an aliquot (whole-cell extract input), the chromatin samples were incubated with antibodies against NF-κB p65 (ab7970 Abcam) or CREB (ab31387) overnight at 4 °C. The samples were then precipitated by binding to protein A-Agarose/Salmon Sperm DNA beads (Millipore, Billerica, MA, USA). The immunoprecipitated chromatin was analyzed using PCR with the primers for the SIRT1 gene promoter and the following cycling parameters: 40 cycles of 58 °C for 60 s and 95 °C for 30 s.

### Tissue preparation and immunohistochemistry

WT and SIRT1 TG mice (8-week-old) were treated with 1 mg/kg Pam3CSK4 for 24 h and then killed. Tissues were fixed through a systemic perfusion, and aortas were dissected carefully and immersed in 10% buffered formalin. Aortic segments were incubated with the anti-rat Mac-1 monoclonal antibody and biotinylated goat anti-mouse IgG, and then reacted with horseradish peroxidase-conjugated streptavidin (Dako). Six to 10 images of each field were captured at various focal lengths using an automatically regulated Z-stepper and Image-Pro4.5J software (Planetron Co., Tokyo, Japan).

Hearts were fixed with 4% paraformaldehyde, dissected, and the proximal half was mounted in OCT compound (Sakura Finetek Inc., Torrance, CA) to prepare the aortic sinus tissue sections. Five-micrometer-thick cross-sections were cut until the aortic sinus appeared. Afterwards, every other section was collected on a slide until the valve cusps were no longer visible. Sections of the aortic sinus were stained with oil red O (Sigma Chemical Co.) to visualize the atherosclerotic lesions. The lesion area was quantified using Carl Zeiss Imaging Systems (AxioVision LE version 4.5, Carl Zeiss Inc., Jena, Germany).

### Statistical analysis

The statistical significance of differences was calculated using one-way ANOVA with the Bonferroni post hoc test for comparisons between multiple groups or Student’s unpaired *t* test for comparisons between two groups. Analyses were performed using GraphPad Prism Software version 5.02 (GraphPad Inc., La Jolla, CA, USA). A *P-*value < 0.05 was considered statistically significant.

## Results

### Involvement of TLR2-induced Mac-1 expression in monocyte adhesion to the endothelium

**Fig. 1:**
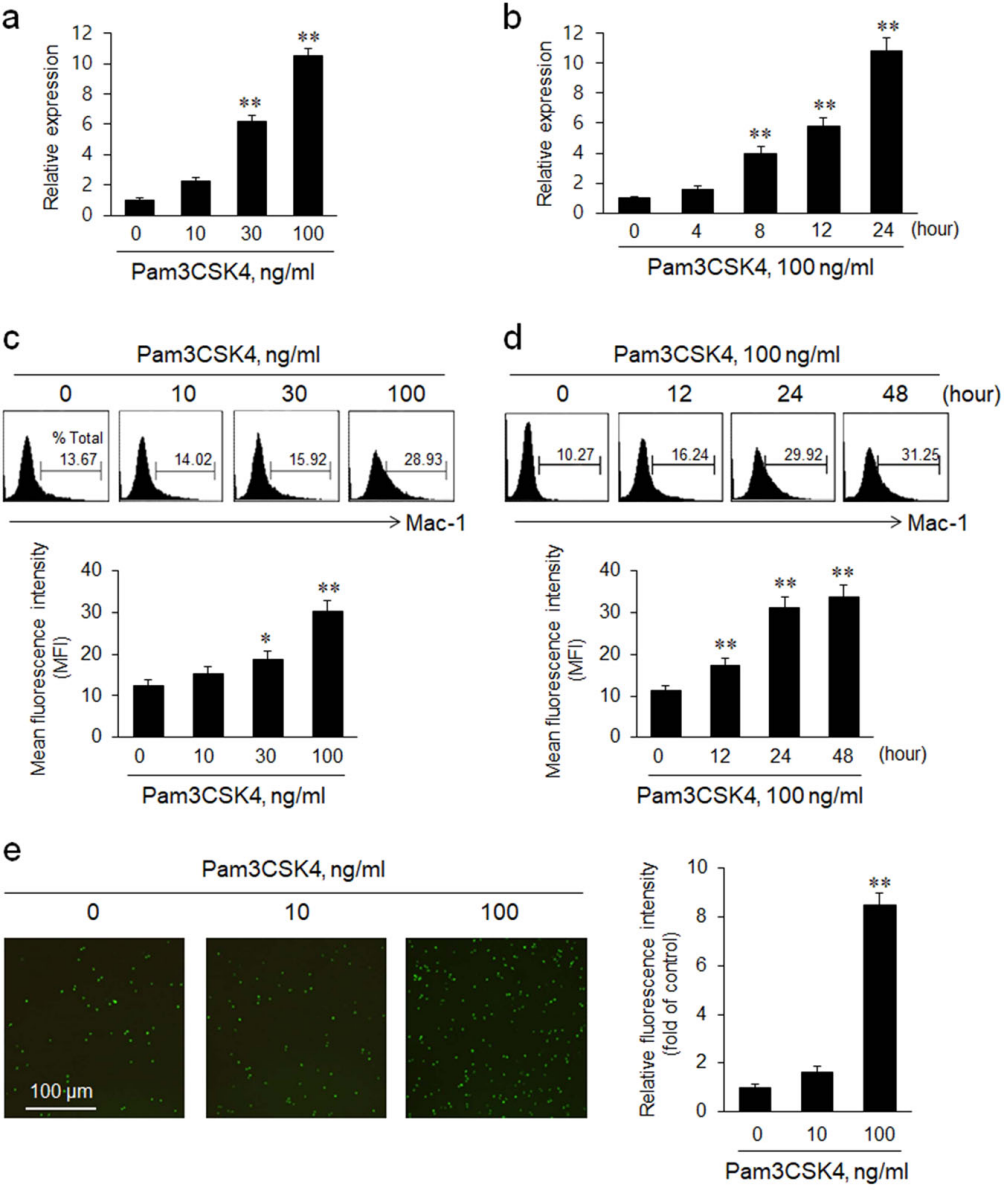
The functional role of TLR2-induced Mac-1 expression in monocyte adhesion to endothelial cells. **a**, **b** THP-1 cells were treated with the indicated concentrations of Pam3CSK4 for 24 h or treated with 100 ng/mL Pam3CSK4 for the indicated times. The total RNA isolated from cells was analyzed for the expression of Mac-1 mRNA using real-time PCR. Quantitative data are presented as the means ± SEM of six independent experiments. **P < 0.01 compared with the control. **c**, **d** Surface expression of Mac-1 protein on THP-1 cells was determined using a phycoerythrin (PE)-conjugated anti-Mac-1 antibody, and presented as mean fluorescent intensities (MFI). Quantitative data are presented as the means ± SEM of seven independent experiments. *P < 0.05 and **P < 0.01 compared with the corresponding control. **e** THP-1 cells were stimulated with the indicated concentrations of Pam3CSK4 for 24 h, labeled with calcein-AM for 30 min, and then co-cultured with HUVECs for 2 h. THP-1 cells that adhered to HUVECs were visualized under a fluorescence microscope. Scale bar = 100 μm. Quantitative data are presented as the means ± SEM of four independent experiments. **P < 0.01 compared with the control

We measured Mac-1 mRNA expression in THP-1 cells stimulated with Pam3CSK4 to determine the effect of TLR2 on Mac-1 expression in THP-1 cells. As shown in Fig. 1a, b, the expression of the Mac-1 mRNA in Pam3CSK4-stimulated THP-1 cells was increased in concentration- and time-dependent manners. Consistent with this upregulation of Mac-1 mRNA expression, the flow-cytometry analysis revealed concentration- and time-dependent increases in levels of the Mac-1 protein in Pam3CSK4-treated cells (Fig. 1c, d). As shown in Fig. 1e, Pam3CSK4-stimulated cells showed increased adhesion to HUVECs in a concentration-dependent manner, confirming the involvement of TLR2-induced Mac-1 expression in monocyte adhesion to endothelial cells.

### Role of SIRT1 in TLR2-induced Mac-1 expression in monocytes

**Fig. 2:**
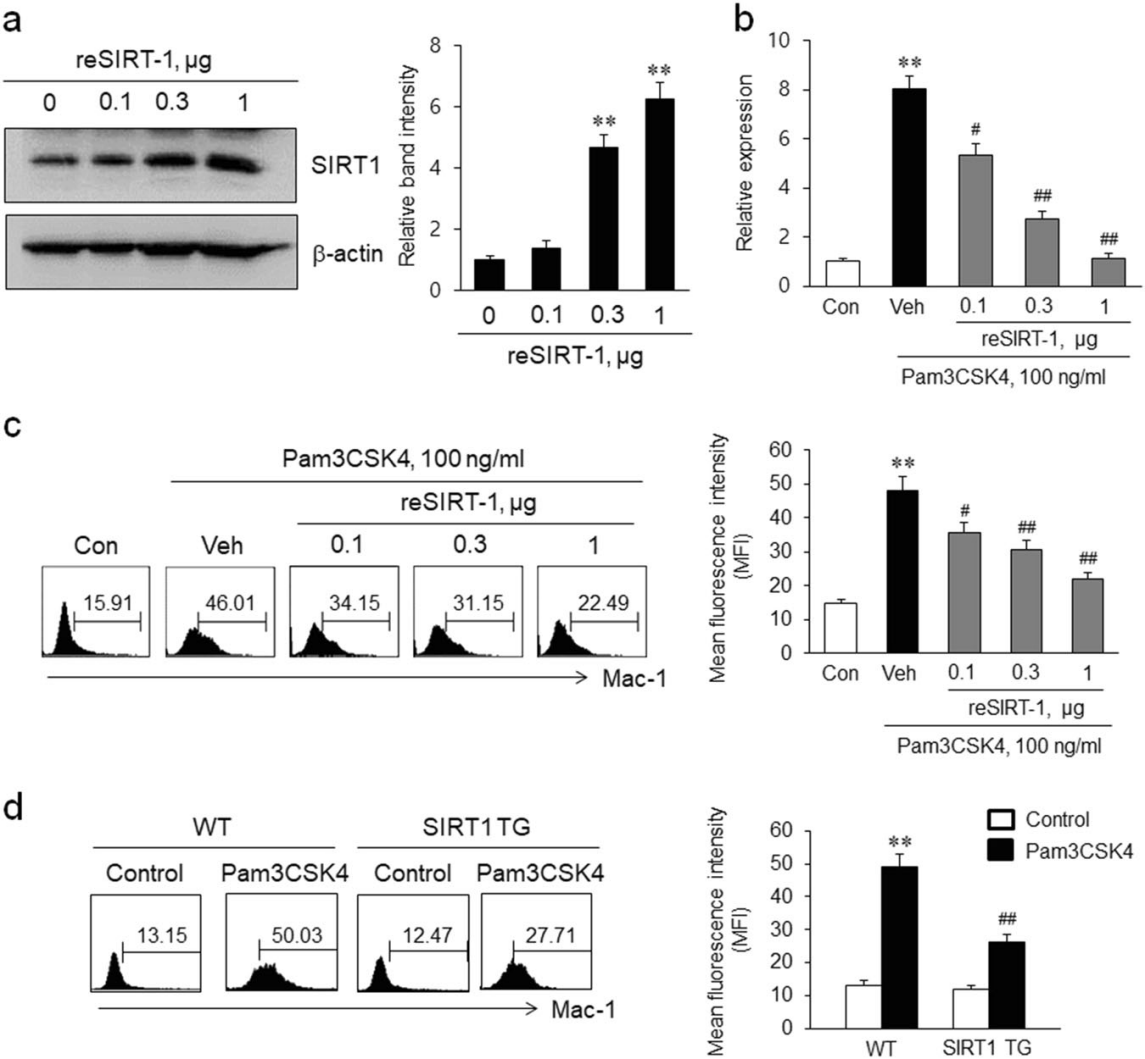
The effects of SIRT1 on TLR2-induced Mac-1 expression in monocytes. **a** THP-1 cells were treated with the indicated concentrations of recombinant SIRT1 (reSIRT1) for 24 h, and cellular SIRT1 levels were determined using Western blotting. Quantitative data are presented as the means ± SEM of four independent experiments. **P < 0.01 compared with the control. **b**, **c** THP-1 cells were pretreated with the indicated concentrations of reSIRT1 for 30 min and then stimulated with 100 ng/mL Pam3CSK4. The levels of Mac-1 mRNA and protein were analyzed using real-time PCR and flow cytometry, respectively. Quantitative data are presented as the means ± SEM of 5–7 independent experiments. **P < 0.01 compared with the control (Con); ^*#*^*P* < 0.05 and ^*##*^*P* < 0.01 compared with the vehicle (Veh). **d** Representative images of flow-cytometry plots of Mac-1 expression in PBMCs isolated from WT and SIRT1 TG mice treated with 1 mg/kg Pam3CSK4 for 24 h. Levels of Mac-1 protein was analyzed using flow cytometry, and mean fluorescent intensities (MFI) were quantified and the data are presented as the means ± SEM of six independent experiments. **P < 0.01 compared with the corresponding control; ^*##*^*P* < 0.01 compared with the corresponding value for WT cells

THP-1 cells were pretreated with reSIRT1 and then stimulated with Pam3CSK4 for 24 h to investigate the effect of SIRT1 on TLR2-induced Mac-1 expression. As shown in Fig. 2a, THP-1 cells treated with reSIRT1 showed an increased level of SIRT1. In cells treated with reSIRT1, Pam3CSK4-induced expression of the Mac-1 mRNA and protein was significantly attenuated in a concentration-dependent manner (Fig. 2b, c). These results were confirmed using PBMCs isolated from WT and SIRT1 TG mice treated with Pam3CSK4. As shown in Fig. 2d, Mac-1 expression was markedly increased in WT PBMCs treated with Pam3CSK4 and significantly attenuated in cells isolated from SIRT1 TG mice.

### Role of SIRT1 in TLR2-induced monocyte adhesion to the endothelium

**Fig. 3:**
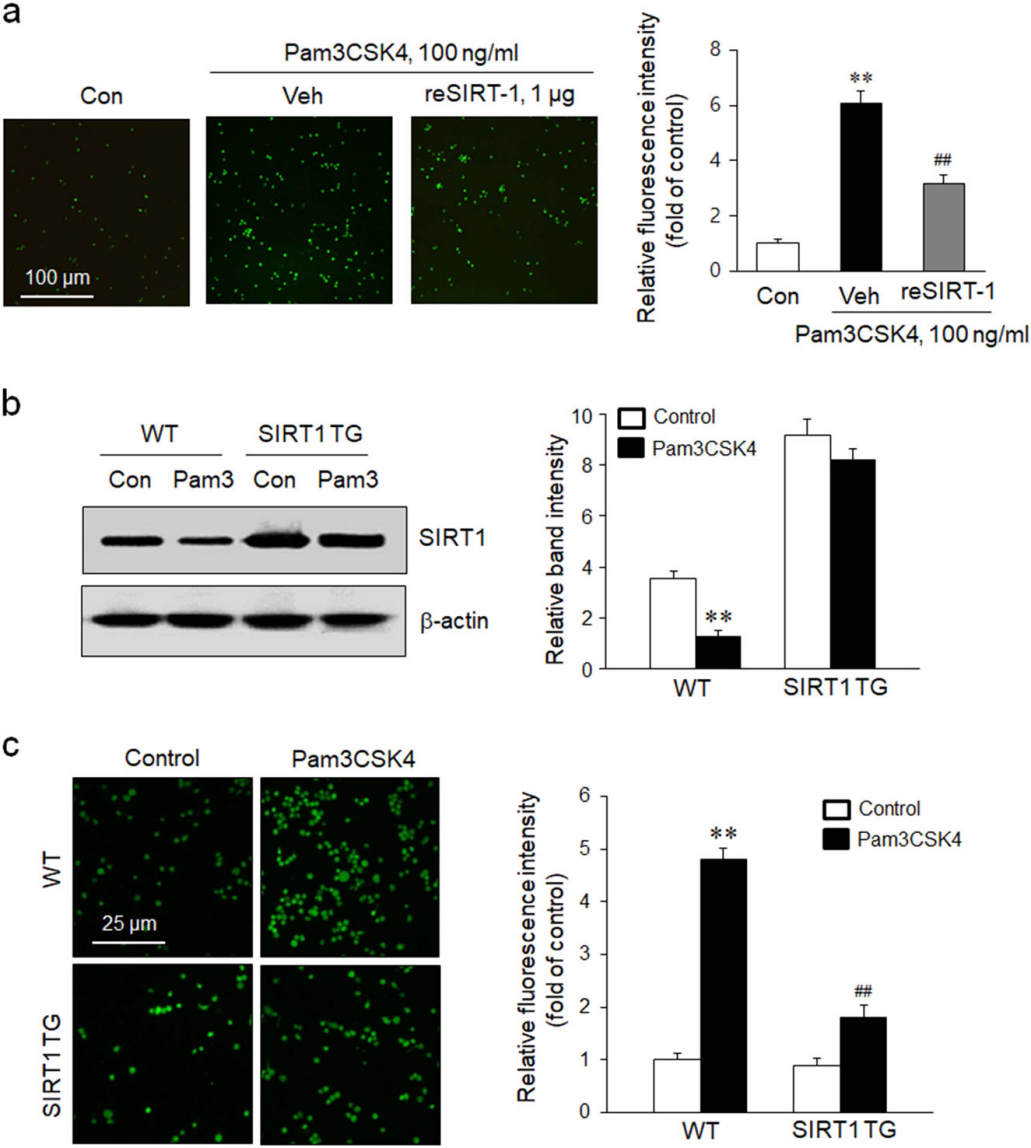
The effects of SIRT1 on TLR2-induced monocyte adhesion to endothelial cells. **a** THP-1 cells were pretreated with recombinant SIRT1 (reSIRT1) for 1 h, stimulated with Pam3CSK4 for 24 h, and then cocultured with HUVECs for 2 h. Cells that adhered to HUVECs were visualized under a fluorescence microscope. Quantitative data are presented as the means ± SEM of six independent experiments. **P < 0.01 compared with the control (Con); ^*##*^*P* < 0.01 compared with the vehicle (Veh). **b** Representative images of immunoblot showing SIRT1 levels in PBMCs isolated from WT and SIRT1 TG mice treated with 1 mg/kg Pam3CSK4 (Pam3) for 24 h. Quantitative data are presented as the means ± SEM of four independent experiments. **P < 0.01 compared with the corresponding control. **c** PBMCs isolated from WT and SIRT1 TG mice treated with 1 mg/kg Pam3CSK4 for 24 h were cocultured with HUVECs for 2 h. Cells that adhered to HUVECs were visualized under a fluorescence microscope, and quantitative data shown in the right panel are presented as the means ± SEM of six independent experiments. **P < 0.01 compared with the corresponding control; ^##^*P* *<* 0.01 compared with the corresponding value for WT cells

The reSIRT1-pretreated THP-1 cells were stimulated with Pam3CSK4 for 24 h and then cocultured with endothelial cells to investigate the effect of SIRT1 on TLR2-induced monocyte adhesion to the endothelium. As shown in Fig. 3a, the endothelial adhesion of monocytes stimulated with Pam3CSK4 was markedly increased, but was significantly attenuated in cells pretreated with reSIRT1. In PBMC of WT mice treated with Pam3CSK4, SIRT1 protein expression was significantly lower than in the control, but was markedly higher in the PBMC of Pam3CSK4-treated SIRT1 TG mice (Fig. 3b). When monocyte adhesion to the endothelium was evaluated using PBMCs isolated from mice, greater endothelial adhesion was observed for PBMCs isolated from Pam3CSK4-treated WT mice than PBMCs isolated from SIRT1 TG mice treated with Pam3CSK4 (Fig. 3c), indicating a pivotal role for SIRT1 in regulating the endothelial adhesion of monocytes.

### Role of TLR2 signaling in regulating SIRT1 expression in monocytes

**Fig. 4:**
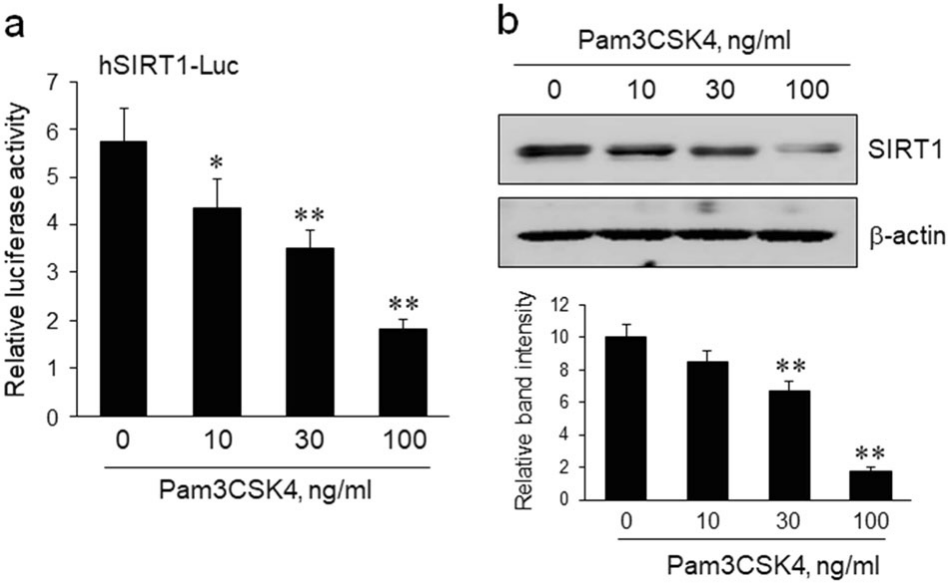
The role of TLR2 signaling in regulating SIRT1 expression in THP-1 cells. **a** THP-1 cells were stimulated with the indicated concentrations of Pam3CSK4 for 24 h, and then the promoter activity of SIRT1 was analyzed by measuring the luciferase reporter activity. Quantitative data are presented as the means ± SEM of six independent experiments. *P < 0.05 and **P < 0.01 compared with the value for concentration 0. **b** Representative images of immunoblots showing the levels of SIRT1 protein in THP-1 cells treated with the indicated concentrations of Pam3CSK4. Immunoblots were quantified, and the data are presented as the means ± SEM of six independent experiments. **P < 0.01 compared with the value for concentration 0

THP-1 cells were stimulated with the indicated concentrations of Pam3CSK4 for 24 h, and then SIRT1 promotor activity and protein expression were determined using a luciferase assay and Western blotting, respectively, to examine the effect of TLR2 signaling on SIRT1 expression in monocytes. As shown in Fig. 4a, the SIRT1 promoter activity was decreased by Pam3CSK4 in a concentration-dependent manner. Likewise, levels of the SIRT1 protein were significantly reduced in cells treated with Pam3CSK4 (Fig. 4b), suggesting a pivotal role for TLR2 signaling in the downregulation of SIRT1 expression in monocytes.

### Cloning and characteristics of the SIRT1 promoter region linked to SIRT1 transcription

**Fig. 5:**
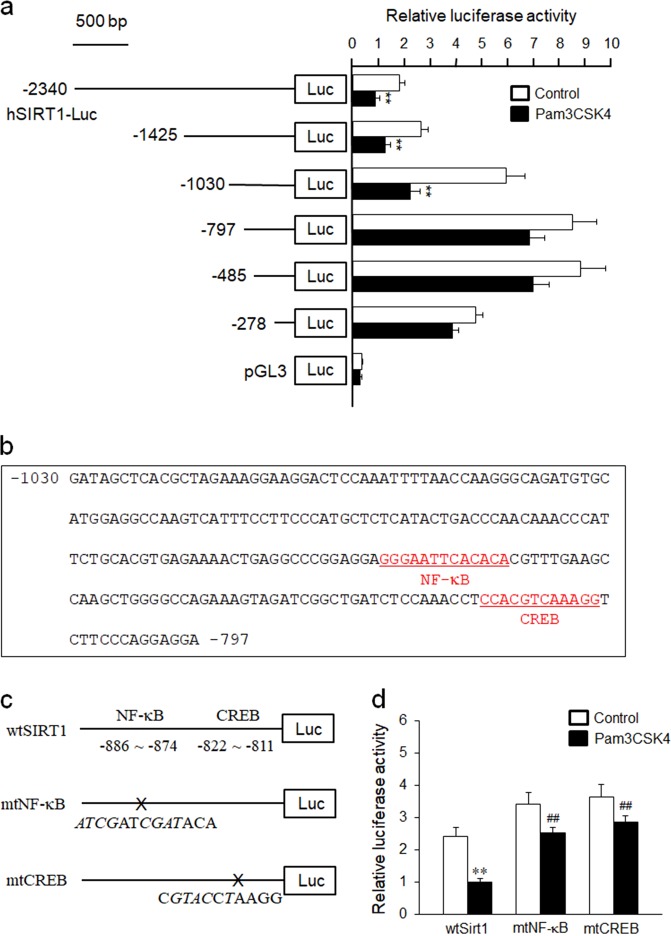
Characteristics of the SIRT1 promoter region involved in Pam3CSK4-mediated downregulation of SIRT1 transcription. **a** THP-1 cells were transiently cotransfected with various promoter constructs and an empty luciferase vector pRL CMV for 24 h, and then stimulated with 100 ng/mL Pam3CSK4 for 24 h. Relative luciferase activities are presented as the means ± SEM of five independent experiments. **P < 0.01 compared with the value for the corresponding control. **b** Nucleotide sequence of the promoter region of the SIRT1 gene. The sequence of the region between nt −1030 and −797 bp of the 5′-flanking region is shown. The underlined sequences are possible transcription factor-binding sites predicted by TFsearch. **c**, **d** THP-1 cells were cotransfected with the empty luciferase vector pRL CMV and promoter constructs containing the WT and mutant constructs of the NF-κB and CREB-binding sites for 36 h (wtSIRT1, wild-type pSIRT1-1030; mtNF-κB, NF-κB mutant; mtCREB, CREB mutant), and then stimulated with 100 ng/mL of Pam3CSK4 for 24 h. Relative luciferase activity is presented as the mean values ± SEM of five independent experiments. **P < 0.01 compared with the corresponding control; ^*##*^*P* < 0.01 compared with the corresponding value for wtSIRT1

As shown in Fig. 5a, the luciferase reporter activity of pSIRT1-2340 after Pam3CSK4 stimulation was 2.0 (±0.2) times lower than the non-stimulated controls. Six promoter constructs of different sizes were prepared and transiently transfected into THP-1 cells, and then luciferase activity was determined to locate the regions within the 2340 nt SIRT1 promoter region responsible for TLR2-mediated suppression of SIRT1 transcription. In response to Pam3CSK4, promoters containing progressive 5′ deletions from nt 2340 to nt 1030 remained strongly inducible. The luciferase reporter activities of Pam3CSK4-stimulated SIRT1-1030 were 2.7 (±0.3) times lower than the corresponding control, suggesting that the region between nt 1030 and nt 797 is responsible for the TLR2-mediated inhibition of SIRT1 promoter activity in THP-1 cells. An analysis of the sequence of the region between nt 1030 and nt 797 revealed the presence of consensus-binding elements for the transcription factors NF-κB and CREB (Fig. 5b).

### Involvement of NF-κB and CREB in the TLR2-mediated suppression of SIRT1 transcription

**Fig. 6:**
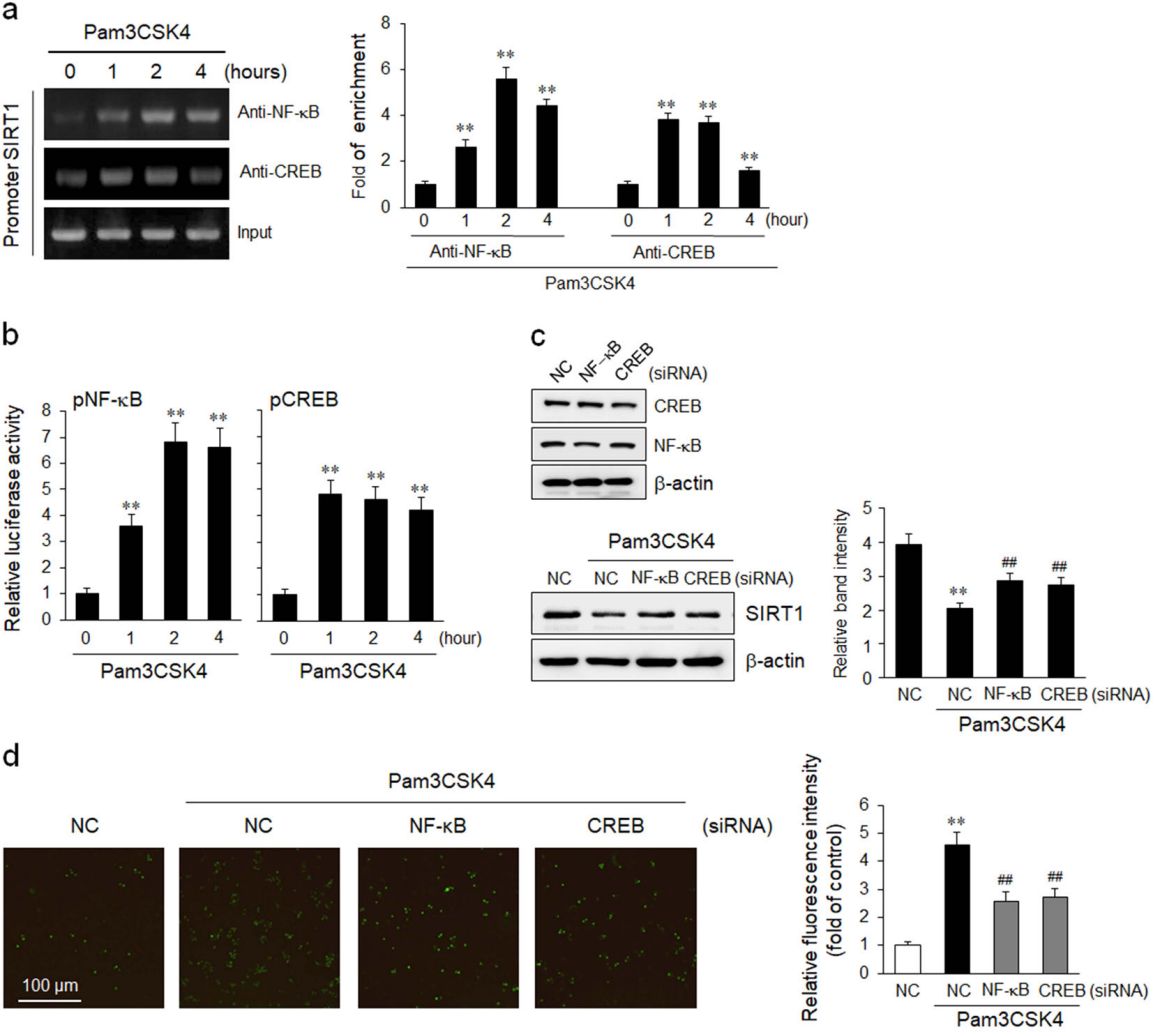
Involvement of NF-κB and CREB in the TLR2-mediated inhibition of SIRT1 transcription in monocytes. **a** The binding of NF-κB and CREB to the SIRT1 promoter was detected using a ChIP assay. NF-κB/DNA and CREB/DNA immune complexes were obtained from THP-1 cells stimulated with Pam3CSK4 for the indicated times. Specific DNA fragments were quantified by PCR, as detailed in the Material and methods section. DNA purified from the lysates of cells incubated without an antibody was used as the input control (Input). Quantitative data are presented as the means ± SEM of six independent experiments. **P < 0.01 compared with the value at time 0. **b** THP-1 cells were transfected with luciferase reporter constructs containing NF-κB and CREB consensus-binding sites and then stimulated with 100 ng/mL of Pam3CSK4 for the indicated times. The quantitation of NF-κB and CREB activities is presented as the means ± SEM of six independent experiments. **P < 0.01 compared with the value at time 0. **c**, **d** THP-1 cells were transfected with NF-κB and CREB siRNAs, and then stimulated with 100 ng/mL Pam3CSK4 for 24 h. SIRT1 expression and cell adhesion to HUVECs were determined. Quantitative data are presented as the means ± SEM of 5–6 independent experiments. **P < 0.01 compared with the negative control (NC); ^*##*^*P* < 0.01 compared with the NC in the Pam3CSK4 group

The predicted NF-κB and CREB-binding sites in the SIRT1 promoter were mutated to examine the roles of NF-κB and CREB in regulating SIRT1 transcription. The SIRT1 promoter activity that was attenuated by Pam3CSK4 was reversed by the mutation of the two consensus sites for NF-κB and CREB (Fig. 5c), indicating that these transcription factors regulated the SIRT1 promoter activity in THP-1 cells (Fig. 5d). Nuclear extracts from Pam3CSK4-stimulated cells were immunoprecipitated with anti-NF-κB or anti-CREB antibodies to assess whether Pam3CSK4 increases NF-κB and CREB binding to sites within the nt 1030 to 797 region of the SIRT1 promoter. Subsequent PCR amplification of the SIRT1 promoter showed increases in NF-κB or CREB binding to the SIRT1 promoter (Fig. 6a). Moreover, Pam3CSK4 significantly increased the activities of NF-κB and CREB (Fig. 6b), indicating the involvement of NF-κB and CREB in the TLR2-mediated suppression of SIRT1 expression. Moreover, both the attenuated SIRT1 expression and the increased endothelial adhesion of THP-1 cells induced by Pam3CSK4 were markedly reversed in NF-κB- or CREB-deficient cells generated using specific siRNAs (Fig. 6c, d).

### Effects of SIRT1 on monocyte adhesion to the vascular endothelium in vivo

**Fig. 7:**
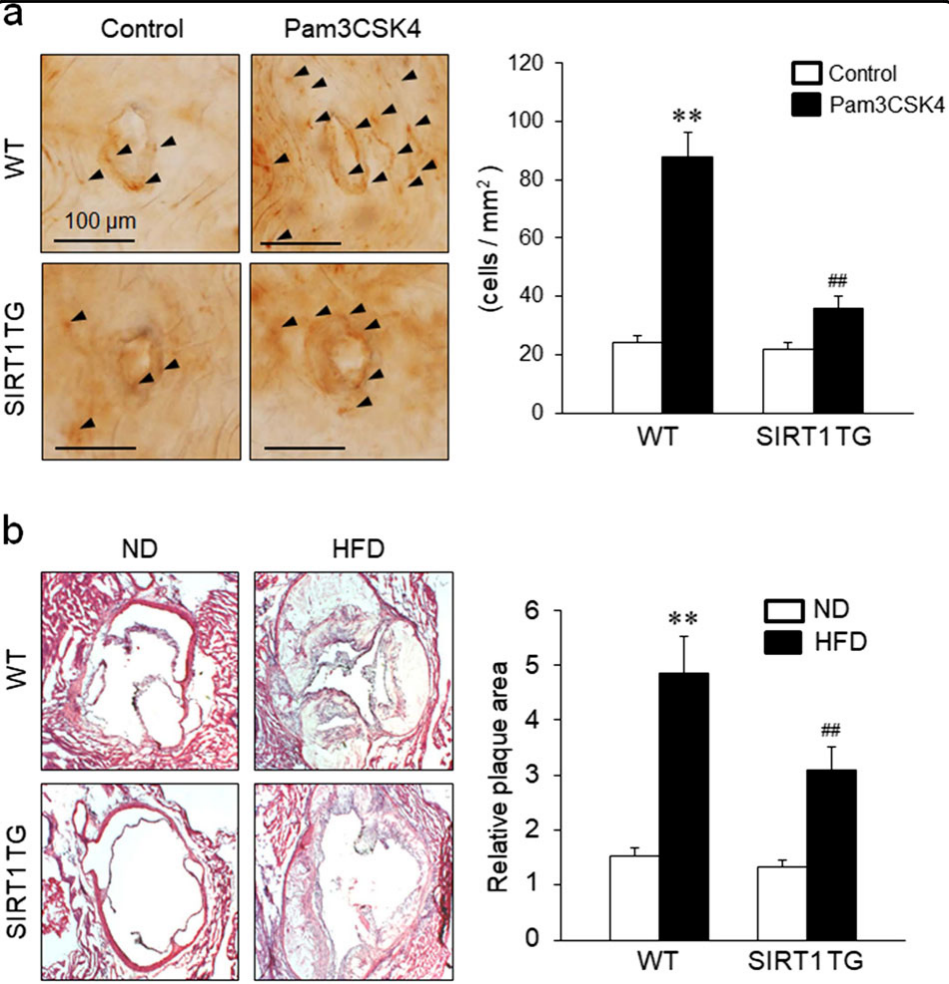
The in vivo effects of SIRT1 on TLR2-mediated monocyte adhesion to the vascular endothelium and atherosclerotic plaque formation. **a** Representative en face views of monocytes adhering to the aortic endothelium of WT and SIRT1 TG mice treated with 1 mg/kg Pam3CSK4 for 24 h. Quantitative data shown in the right panel are presented as the means ± SEM of six independent experiments. **P < 0.01 compared with the corresponding control; ^##^*P* *<* 0.01 compared with the corresponding value for WT mice. Arrowheads denote Mac-1-positive cells. Scale bar = 100 μm. **b** Images of oil red O staining in cross-sections of the aortic sinuses from WT and SIRT1 TG mice fed a high-fat diet (HFD) for 12 weeks. Representative images from five independent experiments are shown. Quantitative data are presented as the means ± SEM of five independent experiments. **P < 0.01 compared with the corresponding control; ^##^*P* *<* 0.01 compared with the corresponding value for WT mice

Using an en face immunohistochemistry method, we determined the effects of SIRT1 on monocyte adhesion to vascular endothelial surfaces surrounding the aortic orifice in vivo. As shown in Fig. 7a, the number of monocytes adhering to the aortic endothelium was significantly increased in WT mice treated with Pam3CSK4 for 24 h, but was significantly decreased in SIRT1 TG mice treated with Pam3CSK4, suggesting that SIRT1 functions as an inhibitor of vascular inflammation. In separate experiments, we determined the role of SIRT1 in atherosclerotic plaque formation as a model of vascular inflammation. As shown in Fig. 7b, atherosclerotic plaques formed in the aortic sinus of WT mice fed a high-fat diet for 12 weeks, but was significantly attenuated in SIRT1 TG mice, suggesting that SIRT1 represents a potential therapeutic target for vascular inflammation, including atherosclerosis, by reducing monocyte adhesion to the vascular endothelium.

## Discussion

In this study, the endothelial adhesion of monocytes stimulated with a TLR2 ligand, Pam3CSK4, was markedly increased in combination with a decrease in SIRT1 expression. The enhanced endothelial adhesion of THP-1 cells induced by Pam3CSK4 was attenuated in cells treated with reSIRT1 and in cells overexpressing SIRT1. Moreover, the *en face* immunohistochemical study showed a marked increase in monocyte adhesion to the aortic endothelium of WT mice treated with Pam3CSK4, but adhesion was significantly attenuated in SIRT1 TG mice, indicating a strong negative relationship between SIRT1 expression and the endothelial adhesion of monocytes.

Monocyte adhesion to the endothelium is a key event leading to vascular inflammation, thus, the events involved in monocyte adhesion to the vascular endothelium might be important therapeutic targets for vascular inflammation, such as atherosclerosis^11^. TLR2 and TLR4 are expressed at high levels in atherosclerotic lesions and associated inflammatory cells, suggesting that TLR2 and TLR4 play dominant roles in vascular inflammation^27^. In our previous study, TLR4 signaling was involved in the development of atherosclerosis in a murine model^28^. In addition, TLR2 is overexpressed in human atherosclerotic plaques and murine models of atherosclerosis^29^. Consistent with previous reports indicating a strong relationship between TLR2 and vascular inflammation, the results of our present study showed a marked increase in the endothelial adhesion of monocytes stimulated with Pam3CSK4, indicating a pivotal role for TLR2 signaling in the progression of vascular inflammation.

Activated monocytes express various adhesion molecules, including β-2 integrins, LFA-1 (CD11a/CD18), Mac-1 (CD11b/CD18), CD11c/CD18, β-1 integrin, and VLA-4 (CD49d/29), which attract and recruit blood monocytes to vessel walls^30,31^. These monocytes then differentiate into macrophages and infiltrate the subendothelial space, where they release and respond to inflammatory mediators, such as tumor necrosis factor-α (TNF-α) and interleukins^32^. Because the interaction between Mac-1 and ICAM-1 is required for the endothelial adhesion of monocytes, Pam3CSK4-induced monocyte adhesion to the endothelium appears to result from the upregulation of Mac-1 on monocytes. In this study, Mac-1 expression was markedly increased in THP-1 cells stimulated with Pam3CSK4 and was attenuated in cells treated with reSIRT1 and in PBMCs isolated from SIRT1 TG mice. In vivo, monocytes adhered to the aortic endothelium of WT mice treated with Pam3CSK4, but adhesion was significantly attenuated in SIRT1 TG mice. Moreover, the increased atherosclerotic plaque formation observed in WT mice fed a high-fat diet was also significantly attenuated in SIRT1 TG mice. Based on these results, SIRT1 plays a pivotal role in preventing vascular inflammation by inhibiting the endothelial adhesion of monocytes through the downregulation of Mac-1 expression. Although atherosclerosis induced by a high-fat diet might not identical to vascular inflammation induced by TLR2, atherosclerosis might be available as a model of TLR2-mediated vascular inflammation because of the strong relationship between TLR2 and atherosclerosis. However, further studies are required to identify the precise role of SIRT1 in TLR2-induced vascular inflammation.

Based on the previous reports describing the transcriptional regulation of SIRT1 expression^33,34^, we postulate that the suppression of SIRT1 expression in cells stimulated with Pam3CSK4 might be controlled at the transcriptional level. We determined the promoter activity and expression of the SIRT1 protein in THP-1 cells stimulated with Pam3CSK4 to investigate the role of TLR2 in regulating SIRT1 expression in monocytes. In this study, the promoter activity and expression of the SIRT1 protein were decreased in cells stimulated with Pam3CSK4, confirming the transcriptional downregulation of SIRT1 expression in monocytes by the TLR2 signaling pathway.

We used a 2.3 kb fragment of the 5′ open-reading frame of SIRT1 (kindly provided by Dr. Masafumi Ito; Gifu International Institute of Biotechnology, Japan) to further identify the regulatory element in the SIRT1 promoter responsible for SIRT1 transcription. The promoter activity of pSIRT1-2034 after Pam3CSK4 stimulation was 2.4 (+0.4) times lower than in untreated control cells, and the promoters containing progressive 5′ deletions from nt 2304 to nt 1030 remained highly inducible in response to Pam3CSK4. Moreover, the promoter activity of Pam3CSK4-stimulated pSIRT1-1030 was 3.8 (+0.6) times lower than untreated controls. Thus, the region between nt 1030 and nt 797 contains binding sites for inhibitory transcription factors regulates the Pam3CSK4-mediated suppression of SIRT1 promoter activity in monocytes. Using the sequence motif search function in TFsearch software (http://mbs.cbrc.jp/research/db/TFSEARCH.html), the putative transcription factor-binding sites are located in the region from −1030 to −797 bp upstream of the transcription initiation site in the SIRT1 promoter. A sequencing analysis confirmed the presence of consensus-binding sites for NF-κB and CREB. In our site-directed mutagenesis study, mutations of the NF-κB and CREB-binding sites in the SIRT1 promoter significantly restored the responsiveness of monocytes to Pam3CSK4. In addition, TLR2-mediated suppression of SIRT1 expression in THP-1 cells was mediated by the transcription factors NF-κB and CREB, suggesting that NF-κB and CREB downregulated for SIRT1 expression in monocytes.

The overexpression of SIRT1 in monocytes has been shown to prevent vascular inflammation by improving vascular function^7,8^. However, the precise contributions of SIRT1 to this process have not been determined. In this study, the increased monocyte adhesion to the vascular endothelium induced by Pam3CSK4 was reversed in cells treated with reSIRT1 and in cells overexpressing SIRT1. Based on the results of our in vivo study, the increased monocyte adhesion to the aortic endothelium of WT mice treated with Pam3CSK4 was significantly attenuated in SIRT1 TG mice, indicating a pivotal role for SIRT1 in preventing monocyte adhesion to the vascular endothelium. Thus, SIRT1 might be a potential target to prevent the progression of vascular inflammatory diseases, including atherosclerosis.
